# Integrated culturomics and 16S rDNA sequencing reveal a functional microbiome signature in endometrial cancer

**DOI:** 10.1186/s12866-026-05242-x

**Published:** 2026-06-10

**Authors:** Teng Liu, Nan Wang, Junkuan Hao, Jiayu Hong, Yanping Han, Lei Yang, Huan Zhang, Jin Zhou, Yafang Tan, Li’an Li, Ruifu Yang

**Affiliations:** 1 Department of Epidemiology and Statistics, School of Public Health, Hebei Medical University, Shijiazhuang, China; 2 State Key Laboratory of Pathogen and Biosecurity, Academy of Military Medical Sciences, Beijing, China; 3 Senior Department of Obstetrics and Gynecology, Chinese PLA General Hospital, Beijing, China; 4 Department of Obstetrics and Gynecology, the First Medical Center, Chinese PLA General Hospital, Beijing, China; 5 Obstetrics and Gynecology, Bethune International Peace Hospital, Shijiazhuang, Hebei China; 6 Department of Parasitology, School of Basic Medical Science, Guizhou Medical University, Guiyang, China; 7 Safety Evaluation Laboratory, Xiyuan Hospital, China Academy of Chinese Medical Sciences, Beijing, China

**Keywords:** Endometrial cancer, Culturomics, 16S rDNA sequencing, Cross-site microbiota, Tumor microenvironment

## Abstract

**Objective:**

Endometrial cancer (EC) is a common gynecologic malignancy globally, but the role of the intratumoral microbiome remains poorly defined. While microbial dysbiosis is increasingly linked to cancer, the composition, function, and clinical relevance of the microbiota in EC are underexplored. This study aims to systematically profile the microbiome in EC patients by integrating culturomics and 16S rDNA sequencing, and to evaluate the functional properties of key bacterial strains in relation to tumor progression.

**Methods:**

We collected cancerous tissues (CATs), non-cancerous adjacent tissues (NATs), and vaginal swabs from 32 EC patients. Culturomics was performed on 57 samples from 19 patients to isolate and identify bacteria, while 16S rDNA sequencing assessed microbial diversity and composition. Functional assays, including flow cytometry for invasion efficiency and ELISA, were used to evaluate pro-inflammatory capacity.

**Results:**

Culturomics identified 79 bacterial species, with *Staphylococcus*, *Streptococcus*, and *Cutibacterium* emerging as core genera shared across the vagina, NATs, and CATs. Functional analysis revealed that strains such as *Staphylococcus epidermidis* and *Streptococcus anginosus* exhibited high invasion efficiency (up to 84.8%) and significantly upregulated pro-inflammatory responses. 16S rDNA sequencing showed that *Lactobacillus* dominated the vaginal microbiota, whereas endometrial tissues were enriched with opportunistic pathogens (e.g., *Stenotrophomonas*). Critically, specific genera were associated with aggressive clinical features: *Slackia* with poorly differentiated (G3) tumors and *Porphyromonas* with deep myometrial invasion (≥ 50%).

**Conclusion:**

This study uncovers a distinct, translocally disseminated core microbiota in EC, with key strains demonstrating invasive and pro-inflammatory capacities that may play a role in tumor progression. These findings provide a foundation for microbial biomarkers and targeted interventions, highlighting the transformative potential of microbiome research in gynecologic oncology.

**Supplementary Information:**

The online version contains supplementary material available at 10.1186/s12866-026-05242-x.

## Introduction

Endometrial cancer (EC) ranks among the most prevalent gynecologic malignancies globally and continues to present substantial clinical challenges [1, 2]. Both its incidence and mortality rates are on the rise annually, with a notable trend towards affecting younger demographics. While established risk factors, including estrogen dysregulation, metabolic abnormalities, and immune dysregulation, are strongly implicated in its pathogenesis, a considerable proportion of cases lack a clear etiology [3]. Emerging evidence indicates that the microbiota influences cancer initiation, progression, and treatment response [4, 5]. The observed association between dysbiosis within the reproductive tract microenvironment and gynecologic tumorigenesis further implies that polymorphic microbiomes may play a previously unexplored role in the development and progression of EC [6, 7].

Through in-depth study of the interaction between microorganisms and hosts, the complex relationship between the gynecological microbiota and EC has gradually been revealed. The vaginal microbiome, particularly non-*Lactobacillus*-dominant communities, is associated with increased EC risk [8, 9]. Similarly, the endometrial microbiota constitutes a functionally active niche [10] and exhibits marked dysbiosis in EC patients [11]. Specific bacteria, such as *Porphyromonas somerae*, demonstrate invasive potential in endometrial cells [12], while *Peptostreptococcus species* may modulate local immune tolerance through metabolites such as indoleacrylic acid [13]. This dysbiosis is closely linked to chronic inflammatory activation, characterized by elevated pro-inflammatory cytokines [14, 15], suggesting that microbiota-driven immunomodulation may foster a pro-carcinogenic microenvironment. Intratumoral microbiota is implicated not only in carcinogenesis but also profoundly influences tumor pathological subtypes and clinical aggressiveness. For instance, in colorectal cancer, enrichment of *Fusobacterium nucleatum* is closely associated with the mesenchymal subtype, which is characterized by heightened invasive and metastatic potential [16]. In pancreatic ductal adenocarcinoma, compositional variations in the intratumoral microbiome can distinguish different tumor immune subtypes and are significantly correlated with shorter patient survival [17, 18]. These microbes directly shape malignant phenotypes through mechanisms such as disrupting intestinal barriers, recruiting immunosuppressive cells, or forming metastatic niches [19, 20]. Therefore, investigating whether and how polymorphic microbiomes in endometrial cancer contribute to the heterogeneity of its histological or molecular subtypes and influence its invasive and metastatic potential is crucial for a deeper understanding of disease progression and prognostic stratification.

Herein, we analyzed specific microbial characteristics in endometrial cancer using multi-site sampling. We performed 16S rDNA sequencing and concurrent bacterial isolation and cultivation on carcinoma tissues, adjacent normal tissues, and vaginal swabs from 32 endometrial cancer patients. We analyzed the global microbial community structure associated with endometrial carcinoma, identified key bacterial strains enriched in carcinoma tissues and validated through cultivation, and revealed their correlations with clinicopathological features. The potentially functionally active strains identified in this study will provide experimental evidence for subsequent mechanistic research on microbe-driven or tumor microenvironment-adapted processes, laying the foundation for exploring microbial biomarkers and intervention strategies for endometrial carcinoma.

## Methods and materials

### Study population

The study was conducted at the Chinese PLA General Hospital. We consecutively enrolled patients initially diagnosed with EC and admitted for surgical treatment. Carcinoma tissues (CATs), non-cancerous adjacent tissues (NATs), and vaginal swabs were collected from these participants. The inclusion and exclusion criteria were as follows: Inclusion Criteria: (1) Patients undergoing surgery with lesion resection in our department and with pathologically confirmed endometrial carcinoma; (2) Complete clinicopathological data available; (3) Agreement to participate with signed informed consent; (4) No antibiotic use within one month; (5) No probiotic consumption within three months. Exclusion Criteria: (1) Receipt of neoadjuvant therapy before surgery; (2) Diagnosis of hereditary endometrial carcinoma (Lynch syndrome or Cowden syndrome).

Clinical findings and samples were collected from 32 patients diagnosed with EC. These patients were further classified histologically into G1 (well-differentiated), G2 (moderately differentiated), and G3 (poorly differentiated) groups.

### Sample collection and preservation

Under sterile conditions during surgical procedures, approximately 1 cm³ specimens of superficial CATs and matched NATs were collected. Each specimen was promptly placed into a pre-filled sterile tube containing PBS. Vaginal specimens were obtained using sterile disposable swabs and eluted in 3 mL of PBS. These samples were kept on ice at 4 °C and transported to the laboratory immediately after the surgical intervention. Half of each tissue sample was thoroughly homogenized, and 1 mL of PBS from the vaginal swabs was collected for primary cell isolation and culture under specified conditions. The remaining half was stored at -80 °C for subsequent analyses. The detailed workflow of sample collection and processing is shown in Figure S1.

### Microbial culturomics

Microbial isolation was performed aseptically using endometrial tissue and vaginal swab samples obtained from 19 EC patients. Tissue samples were minced and homogenized in sterile PBS, and 1 mL of PBS eluent from vaginal swabs was used directly. Both tissue homogenate and swab eluent (1 mL each) were inoculated into aerobic and anaerobic blood culture bottles containing 5 mL of sheep blood. Anaerobic bottles were placed in an anaerobic bag, and all bottles were incubated at 37 °C for up to 15 days. Subculturing was performed on days 0, 3, 7, and 15 by spreading 100 µL of broth onto Columbia blood agar plates. Tissue samples were plated directly; swab samples were serially diluted in PBS before plating. Plates were incubated aerobically at 37 °C for 24 h or anaerobically at 37 °C for 72 h. After incubation, distinct colonies were selected and purified on Columbia blood agar. A pure colony from each was grown in Brain Heart Infusion broth under specific conditions (24 h aerobic or 72 h anaerobic). For long-term storage, cultures were mixed with 50% glycerol and stored at -80 °C.

Colonies picked after cultivation were identified using the MALDI-TOF mass spectrometry system (Autof MS1000). Colonies not identifiable via the MALDI-TOF mass spectrometry database V1.1.12 (score ≥ 9) underwent 16S rRNA gene sequencing with primers 27 F (59’-agagtttgatcmtggctcag-39’) and 1492R (59’-ggttaccttgttacgactt-39’). Sequence similarity analysis was performed using the NCBI BLAST algorithm against reference strains. Strains exhibiting > 98.65% similarity to a reference strain were classified as belonging to the same species.

### Core taxa definition

To identify core genera, all recovered genera from all samples were included irrespective of their anatomical distribution. The discrete random expectation was calculated for each genus based on its isolation frequencies across vaginal swabs, NATs, and CATs. Genera with expectation values above the 75th percentile determined by quartile analysis were defined as core genera.

For cross-site core taxa at the species level, only species detected in at least two anatomical sites were included to exclude rare sporadic isolates with low biological representativeness. After calculating their discrete random expectation values, species exceeding the 75th percentile were defined as cross-site core species.

### FISH

Fresh tissue samples were rinsed with PBS, fixed in 4% paraformaldehyde at 4 °C for over 12 h, dehydrated in ethanol, cleared in xylene, and embedded in paraffin. Sections were cut to 4 μm, mounted on slides, baked at 62 °C for 2 h, deparaffinized, and rehydrated. They were digested with proteinase K, washed in PBS, pre-hybridized, and hybridized overnight at 40 °C with a probe. Post-hybridization washes were performed with SSC solutions. Sections were blocked with 3% BSA, counterstained with DAPI, and mounted with anti-fade medium. Imaging was performed on a Nikon fluorescence microscope (Nikon DS-U3, Japan).

### DNA extraction

Genomic DNA was extracted from tissue samples and vaginal swabs using the cetyltrimethylammonium bromide (CTAB) method. Samples were lysed with CTAB buffer and lysozyme at 65 °C, then centrifuged to collect the supernatant. Nucleic acids were extracted with phenol: chloroform: isoamyl alcohol, followed by chloroform: isoamyl alcohol, each followed by centrifugation. The aqueous phase was transferred, and DNA was precipitated with isopropanol at -20 °C. The DNA pellet was collected by centrifugation, washed with 75% ethanol, air-dried, and resuspended in ddH₂O.

### 16S rDNA sequencing

The 16S rDNA V3-V4 region was selected for PCR amplification (primers: 341 F, 59-CCTAYGGGR BGCASCAG-39; 806R, 59-GGACTACNNGGGTATCTAAT-39). Sequencing libraries were prepared using a TruSeq DNA PCR-free sample preparation kit (Illumina, USA). Library quality was assessed using Qubit and Q-PCR. Sequencing was performed on an Illumina NovaSeq 6000 platform (Illumina, San Diego, CA, USA). 

### Sequence analysis

Raw sequences were spliced and quality-controlled to produce clean tags. Chimera filtering was then applied to generate effective tags for further analysis. Effective tags were denoised in QIIME2 (version QIIME2-202202) to generate representative sequences for each ASV. ASVs were annotated to species using the Silva 138.1 database. *α*-diversity was assessed using the Shannon, Simpson, and Chao1 indices; *β*-diversity was calculated using Bray-Curtis distances and visualized by principal-coordinate analysis (PCoA). LEfSe identified key microbial features that distinguished groups, and ANOSIM confirmed significant inter-group differences relative to intra-group variation.

### Bacterial invasion assay

Bacterial strains stored in glycerol were reactivated by overnight shaking at 37 °C, then 50–100 µL was transferred to fresh BHI medium for cultivation. Based on growth rates, bacteria were adjusted to an OD_600_ of 1.0, centrifuged at 4500 rpm for 5 min, and resuspended in PBS containing 1 µg/mL of 5-(and 6)-carboxyfluorescein diacetate succinimidyl ester (CFSE) (Invitrogen, Thermo Fisher Scientific Inc., Waltham, MA, USA). After a 30-minute incubation at 37 °C with intermittent shaking, the mixture was centrifuged at 4500 rpm for 5 min, and the procedure was repeated three times to remove excess dye. Simultaneously, Ishikawa cells at 80% confluency were trypsinized, neutralized with DMEM and 10% FBS, centrifuged, counted, and seeded into 6-well plates at 2 × 10⁶ cells per well, then incubated overnight at 37 °C with 5% CO_2_. CFSE-labeled bacteria were used to infect cells at a 10:1 bacteria-to-cell ratio, and the cells were then centrifuged at 1000 rpm for 5 min. Aerobic bacteria were incubated at 37 °C with 5% CO_2_ for 2 h, whereas anaerobic bacteria were incubated in anaerobic bags for the same period. Following incubation, 200 µg/mL of gentamicin was added for 60 min to eradicate extracellular bacteria. The media was discarded, and the cells were rinsed 5 times with PBS containing 1% serum. Flow cytometry (Sony MA900, Sony Biotechnology) was used to quantify the proportion of CFSE-labeled bacteria that infiltrated Ishikawa cells, and analyzed with FlowJo software.

### Cytokine ELISA assays

Collect the supernatant after a 6-hour co-incubation of bacteria with THP-1 cells. Quantify TNF-α and IL-1β concentrations using ELISA kits (Dakewe Biotech Co., China) by following these steps: Add 100 µL of standards, controls, and supernatant to the pre-coated plates. Incubate for three hours at ambient temperature after adding 50 µL of detection antibody. Wash three times, then add 100 µL of enzyme conjugate and incubate for 20 min. Wash three more times, then add 100 µL of substrate solution and incubate in the dark for 30 min. Stop the reaction with 100 µL of the stop solution, then measure the absorbance at 450 nm. Determine cytokine concentrations using a standard curve generated from recombinant TNF-α and IL-1β.

### Statistics

Statistical analyses were conducted using R and SPSS version 26.0. The chi-square test was used to evaluate the clinical findings, and the t-test and Wilcoxon test were applied to assess intergroup differences in sample diversity indices. One-way ANOVA and Kruskal–Wallis test were used to evaluate differences among multiple groups, and Tukey’s test was performed for pairwise comparisons. Discrete random expectation and quartile calculation were used to identify cross-site core taxa, and the quartile method was applied to evaluate bacterial invasion efficiency. A *P*-value of less than 0.05 was considered statistically significant.

## Results

### Clinical observations

The study included 32 individuals who were histologically graded as G1 (*n* = 12), G2 (*n* = 15), and G3 (*n* = 5). Among them, 83.3% of the G1 group were classified as stage IA, indicating a high prevalence of early-stage disease. Stage IA was noted in 46.7% of the G2 group and 40% of the G3 group. Advanced stages (II and III) were primarily observed in the G2 and G3 groups. Marked age disparities were observed among the groups: G2 and G3 patients were older (61.80 ± 9.09 and 60.20 ± 7.53 years, respectively) than those in G1 (47.58 ± 7.55 years, *P* < 0.05). Increased myometrial invasion (≥ 50%) was associated with higher pathological grade, observed in 46.7% of G2 and 60% of G3 patients, compared with 8.3% in G1 patients. Lymphovascular space invasion (LVSI) was identified exclusively in G3 (40%). Diabetes was markedly correlated with G2 (40%), but was absent in G1 and G3 (Table 1). Other factors showed no notable differences across grades (Table S1).

**Table 1:** Clinical characteristics across different pathological groups

Category(Total = *N*/%)	Histological grade			*P*-value
	G1(*N* = 12)	G2(*N* = 15)	G3(*N* = 5)	
FIGO stage				
IA	10(83)	7(46.7)	2(40)	
IB	1(8.3)	5(33.3)	1(20)	
II	0(0)	3(20)	1(20)	
IIIA	1(8.3)	0(0)	0(0)	
IIIB	0(0)	0(0)	1(20)	
Age(Mean ± SD)	47.58 ± 7.55 ^b^	61.80 ± 9.09 ^a^	60.20 ± 7.53 ^a^	0
BMI(Mean ± SD)	26.88 ± 4.37	24.35 ± 3.71	22.50 ± 2.02	0.445
Tumour size(cm)				0.107
< 3.0	9(75)	5(33.3)	3(60)	
>=3.0	3(25)	10(66.7)	2(40)	
With squamous differentiation (5/13.5)	2(16.67)	1(6.7)	2(40)	0.214
LVSI (2/5.4)	0 (0) ^a^	0 (0) ^a^	2(40) ^b^	0.004
Myometrial invasion				0.029
< 50%(26/70.3)	11 (91.7) ^b^	8 (53.3) ^a^	2(40) ^a^	
>=50%(11/29.7)	1 (8.3) ^a^	7 (46.67) ^a^	3(60) ^a^	
Cervical stromal invasion (3/8.1)	0 (0)	2 (13.3)	1(20)	0.392
with chronic cervicitis (32/86.5)	11(91.7)	13(86.7)	3(60)	0.255
with retention cyst (13/35.1)	4(33.3)	4(26.7)	1(20)	0.144
with Diabetes (8/21.6)	0(0)	6 (40)	0(0)	0.034
with Hypertension (13/35.1)	3 (25)	7(46.7)	1(20)	0.293
with myomectomy (15/40.5)	3 (25)	7(46.7)	2(40)	0.528
with adenomyosis(5/13.5)	2(16.7)	3(20)	0(0)	0.551

Different superscript letters indicate significant differences. *P* values were calculated by the chi-square test. *P* < 0.05

### Isolation and identification of bacteria in endometrial tissue and vaginal swabs

**Fig. 1:**
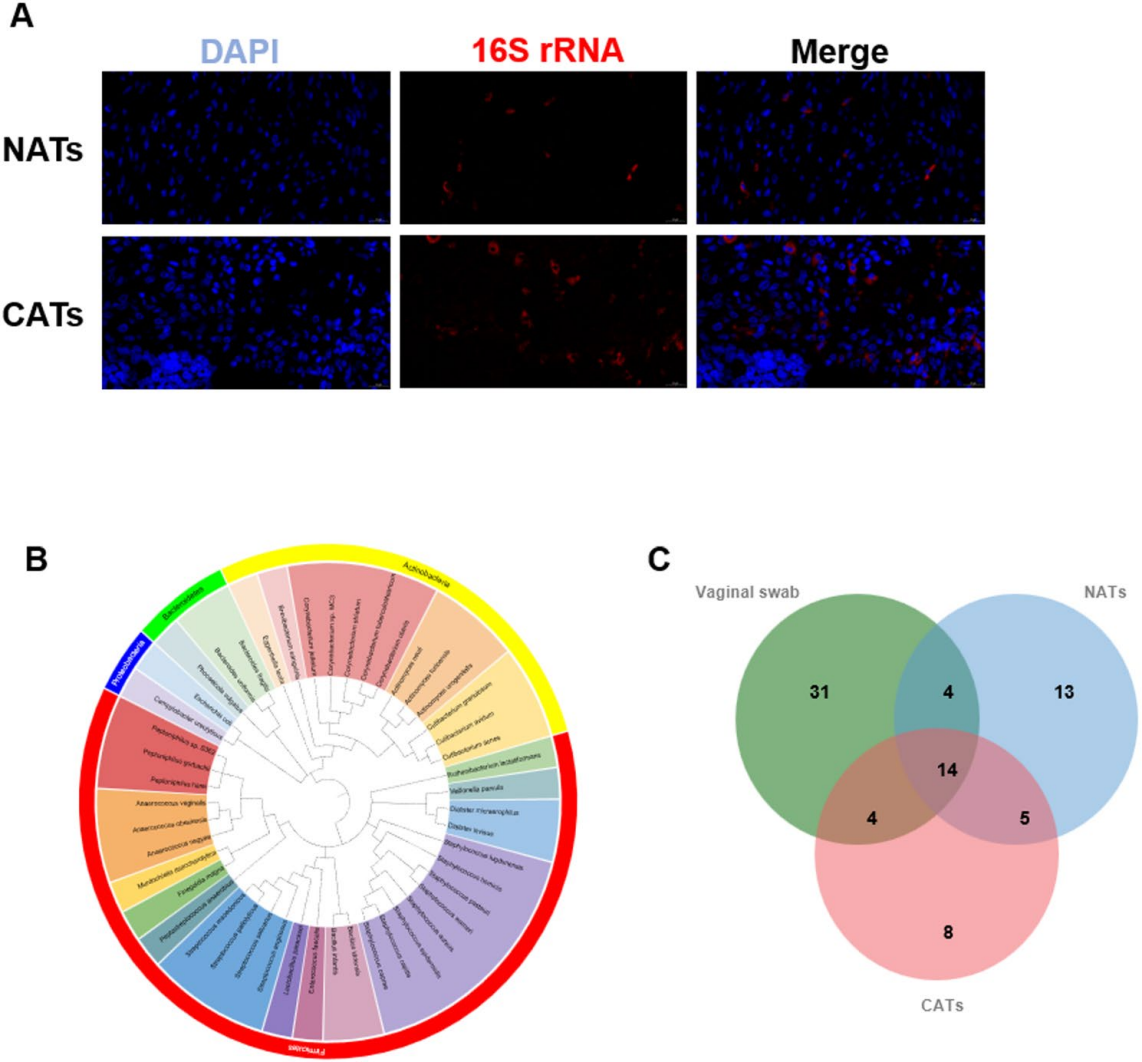
Bacterial localization and identification in tissue samples. **A** FISH analysis of bacterial 16S rRNA in a tissue sample stained with the EUB388 probe (red) and DAPI (blue), images were photographed at *400x* magnification. The scale bar distance (20 μm) is indicated on the figure. **B** Phylogenetic tree and proportions of 47 bacterial species isolated from the tissue samples. **C** Venn diagrams of 79 bacterial species across all three sample types

To investigate the bacterial community in endometrial cancer, we initially employed FISH to detect bacteria in tissues, observing elevated signals in CATs compared with NATs (Fig. 1A). We subsequently cultured 57 samples from 19 patients, including vaginal swabs, NATs, and CATs, and identified 479 bacterial colonies belonging to 79 species via MALDI-TOF or 16S rRNA sequencing. Analysis of tissue samples revealed 152 colonies (84 from CATs, 68 from NATs) represented 47 species (31 from CATs, 36 from NATs) across four phyla: *Firmicutes* (61.7%), *Actinobacteria* (27.7%), *Bacteroidetes* (6.4%), and *Proteobacteria* (4.3%) (Fig. 1B). Vaginal swabs exhibited a comparable phylum composition (Figure S2).

The Venn diagram shows that among the 79 bacterial species, 14 were isolated from all three sample types, while 31, 13, and 8 were isolated exclusively from vaginal swabs, NATs, and CATs, respectively. Species isolated from two sample types included 5 common to NATs and CATs, 4 common to vaginal swabs and NATs, and 4 additional species common to vaginal swabs and CATs (Fig. 1C).

14 bacterial species showed high isolation frequencies across all three sample types. Among these, *Staphylococcus epidermidis* had the highest isolation frequency in vaginal swabs (84.2%) and CATs (26.3%), whereas *Finegoldia magna* had the highest isolation frequency in NATs (26.3%). *Staphylococcus epidermidis*, *Streptococcus anginosus*, *Finegoldia magna*, *Enterococcus faecalis*, *Escherichia coli*, *Staphylococcus lugdunensis*, and *Cutibacterium acnes* were identified as cross-site core species with random expectation ≥ 44.5% (75th percentile, Table 2). Only 8 species isolated from the CATs site showed low isolation frequencies; except for *Staphylococcus capitis* (10.5%), all others provided only 1 isolate each (5.3%) (Table S2). Among the species common to both sites, *Peptoniphilus harei* was isolated from vaginal swabs at a frequency of 31.6%, and one strain was obtained in NATs. *Anaerococcus vaginalis* was isolated from vaginal swabs at a frequency of 21.1%, and one strain was cultured in CATs. The isolation frequencies of the other bacterial species across two sample types were relatively low (Table S3).

**Table 2:** Isolation frequency of species common to all three sample types

Species	Frequence of detection (%)			Random expectation (%)
	Vaginal swab(*N* = 19)	NATs(*N* = 19)	CATs(*N* = 19)	
*Staphylococcus epidermidis*	84.2	21.1	26.3	90.8
*Streptococcus anginosus*	78.9	15.8	15.8	85
*Finegoldia magna*	63.2	26.3	21.1	78.6
*Enterococcus faecalis*	63.2	10.5	26.3	75.7
*Escherichia coli*	36.8	15.8	26.3	60.8
*Staphylococcus lugdunensis*	42.1	15.8	10.5	56.4
*Cutibacterium acnes*	26.3	10.5	15.8	44.5
*Cutibacterium avidum*	21.1	10.5	21.1	44.3
*Actinomyces urogenitalis*	26.3	5.3	5.3	33.9
*Staphylococcus hominis*	15.8	5.3	10.5	28.6
*Streptococcus gallolyticus*	15.8	5.3	5.3	24.5
*Corynebacterium tuberculostearicum*	10.5	5.3	10.5	24.1
*Corynebacterium striatum*	10.5	5.3	5.3	19.7
*Dialister micraerophilus*	5.3	5.3	5.3	15.1

At the genus level, *Staphylococcus exhibited* the highest isolation frequency across all three sample types, and the isolation frequency of *Cutibacterium* in CATs only lower than *Staphylococcus*. 9 genera with random expectation ≥ 49.6% (75th percentile) were identified as core genera, including *Staphylococcus*, *Streptococcus*, *Finegoldia*, *Enterococcus*, *Corynebacterium*, *Escherichia*, *Cutibacterium*, *Actinomyces*, *Peptoniphilus* (Table 3).

**Table 3:** Isolation frequency of bacterial genera from different sample types

Genus	Frequence of detection (%)			Random expectation (%)
	Vaginal swab(*N* = 19)	NATs(*N* = 19)	CATs(*N* = 19)	
*Staphylococcus*	89.5	36.8	42.1	96.2
*Streptococcus*	73.7	15.8	21.1	82.5
*Finegoldia*	63.2	26.3	21.1	78.6
*Enterococcus*	57.9	10.5	26.3	72.2
*Corynebacterium*	47.4	21.1	21.1	67.3
*Escherichia*	31.6	15.8	26.3	57.6
*Cutibacterium*	21.1	15.8	31.6	54.6
*Actinomyces*	36.8	5.3	15.8	49.6
*Peptoniphilus*	36.8	15.8	5.3	49.6
*Anaerococcus*	26.3	15.8	5.3	41.2
*Klebsiella*	36.8	0	0	36.8
*Lactobacillus*	21.1	5.3	5.3	29.2
*Bacteroides*	10.5	10.5	5.3	24.1
*Dialister*	5.3	5.3	10.5	19.7
*Arthrobacter*	15.8	0	0	15.8
*Facklamia*	15.8	0	0	15.8
*Actinotignum*	10.5	0	0	10.5
*Bacillus*	0	5.3	5.3	10.3
*Veillonella*	0	0	5.3	5.3
*Brevibacterium*	0	0	5.3	5.3
*Collinsella*	5.3	0	0	5.3
*Flavonifractor*	5.3	0	0	5.3
*Clostridium*	5.3	0	0	5.3
*Microbacterium*	5.3	0	0	5.3
*Kocuria*	5.3	0	0	5.3
*Ralstonia*	5.3	0	0	5.3
*Parabacteroides*	5.3	0	0	5.3
*Morganella*	5.3	0	0	5.3
*Campylobacter*	0	5.3	0	5.3
*Peptostreptococcus*	0	5.3	0	5.3
*Murdochiella*	0	5.3	0	5.3
*Eggerthella*	0	5.3	0	5.3
*Alloiococcus*	5.3	0	0	5.3
*Slackia*	5.3	0	0	5.3
*Prevotella*	5.3	0	0	5.3

While results from culture isolation and 16S rDNA sequencing were not fully consistent due to methodological differences. Further analyses will be performed to interpret these discrepancies in our subsequent work.

### Microbial composition and diversity of endometrial and vaginal microbiota

**Fig. 2:**
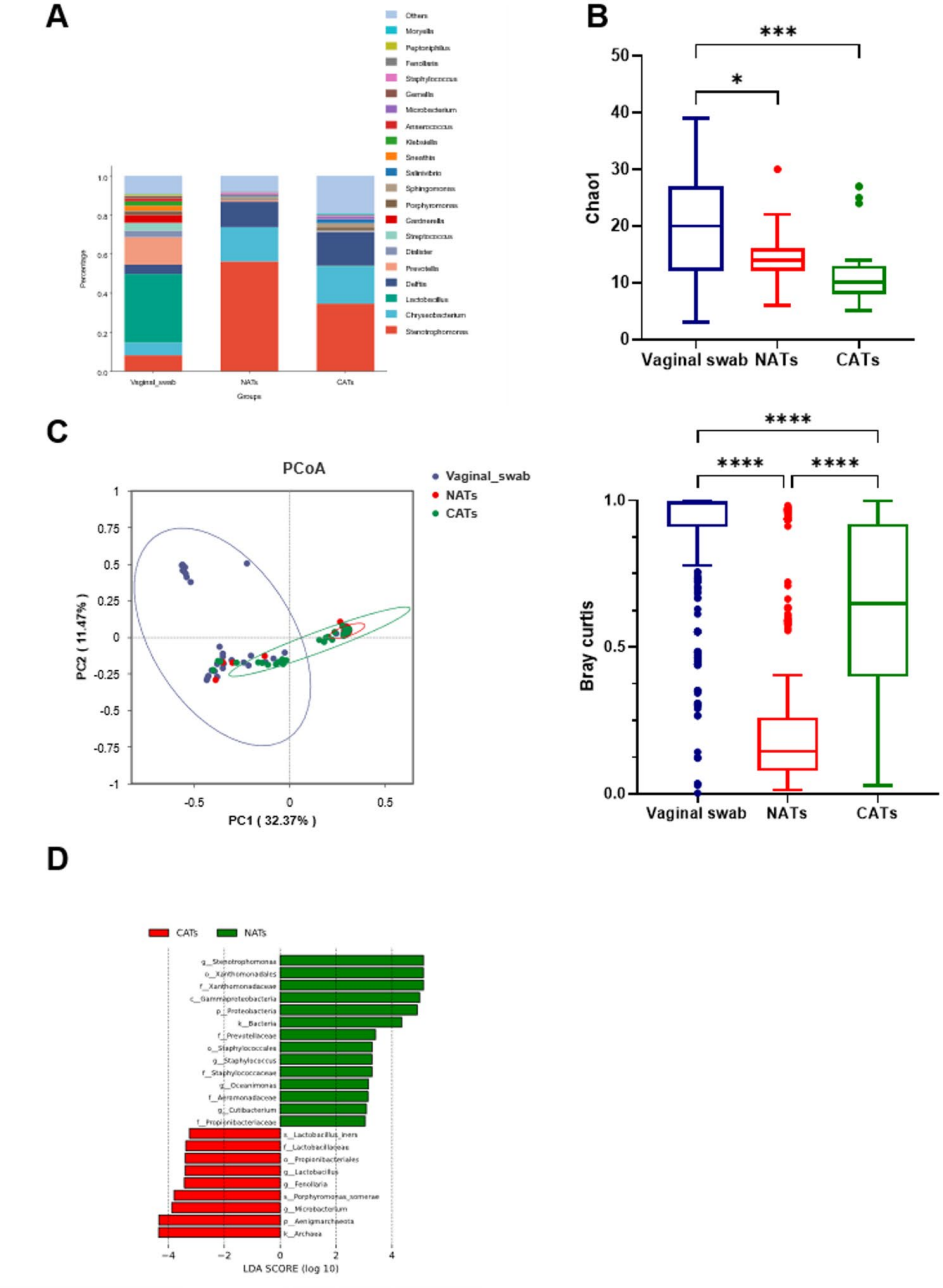
Endometrial and vaginal microbiota composition and diversity. **A** Taxonomic composition at the genus level in endometrial and vaginal samples, based on 16S rRNA gene amplicon sequencing (top 20). **B** Chao1 diversity index for endometrial and vaginal samples. *P* values were calculated using the Wilcoxon test. *, *P* < 0.05; ****, *P* < 0.0001. **C** Bray-Curtis *β*-diversity distance matrix presented as a box plot and PCoA. *P* values were calculated using the Wilcoxon test. ****, *P* < 0.0001. **D** LEfSe was used to identify bacterial taxa that differed significantly between NATs and CATs. Only taxa with LDA > 3 and *P* < 0.05 are shown

To elucidate microbial community characteristics, we performed 16S rDNA sequencing on vaginal swabs and tissue specimens. Vaginal samples were primarily dominated by *Lactobacillus*, with *Prevotella*, *Gardnerella*, and *Stenotrophomonas* as secondary taxa. In contrast, tissue samples showed no dominance by *Lactobacillus*; instead, *Stenotrophomonas* was prevalent, and *Chryseobacterium* and *Delftia* were present in varying amounts (Fig. 2A). The *α*-diversity analysis showed that the Chao1 index for CATs was significantly lower than for NATs and Vaginal swabs, indicating a difference in species richness (*P* < 0.05; Fig. 2B). However, the Shannon and Simpson indices did not differ significantly among groups (Figure S3). *β*-diversity analysis using Bray-Curtis distances revealed a statistically significant difference in the overall microbiota structure among the vaginal, NATs, and CATs groups (*P* < 0.05; Fig. 2C). LEfSe analysis identified differentially enriched bacterial genera between NATs and CAT: *Lactobacillus*, *Fenollaria*, and *Microbacterium* were significantly enriched in CATs, while *Stenotrophomonas*, *Staphylococcus*, *Oceanimonas*, and *Cutibacterium* were enriched in NATs (Fig. 2D).

### Different pathogenic strains shape distinct uterine microbiomes

**Fig. 3:**
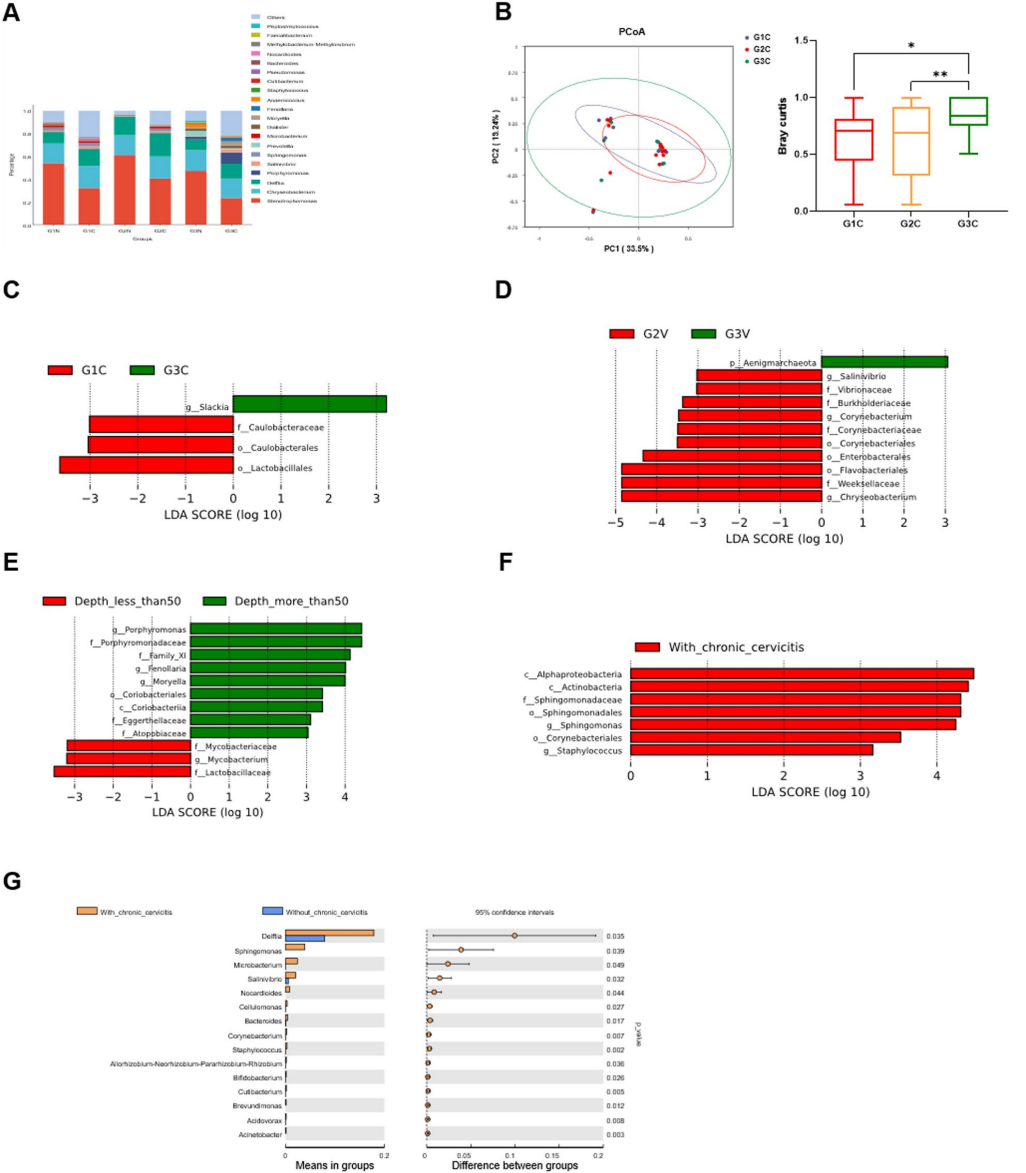
Characterization of microbiotas across histological grades and clinical features. (**A**) Taxonomic composition at the genus level for histological grades in NATs and CATs, based on 16S rRNA gene amplicon sequencing (top 20). (**B**) Bray-Curtis β-diversity distance matrix for histological grade in CATs, shown as a box plot and PCoA. LEfSe was used to identify bacterial microbiota that differed significantly among histological grades in CATs (**C**) and in the vagina (**D**). LEfSe was also used to identify bacterial microbiota that differed significantly by depth of myometrial invasion (**E**) and by the presence or absence of chronic cervicitis (**F**). Only taxa with a significant LDA > 3 and a P value < 0.05 are shown. (**G**) Bacterial taxa with significantly different relative abundances from patients with vs. without chronic cervicitis. *P* values were calculated using the Wilcoxon test. *, *P* < 0.05; **, *P* < 0.01

Microbial profiles were examined across histological grades and clinical features. CATs consistently showed higher species richness than paired NATs across all grades. The dominant genera across all groups were *Stenotrophomonas*, *Delftia*, and *Chryseobacterium*. *Porphyromonas* abundance increased in G3, especially in CATs (Fig. 3A). Within CATs (Fig. 3B) and in the Vagina (Figure S4), *β*-diversity was significantly higher in G3 than in G1 and G2, as measured by Bray-Curtis distances (*P* < 0.05). In contrast, no significant differences in *β*-diversity were observed among histological grades in NATs (Figure S3). Moreover, *α*-diversity indices did not differ significantly in CATs across histological grades (Figure S5).

LEfSe analysis identified taxonomic enrichment patterns across histological grades and sample types. In the vagina, G2 showed significant enrichment of the taxa *Chryseobacterium*, *Corynebacterium*, and *Salinivibrio* (Fig. 3D). In CATs, the *Slackia* genus was significantly enriched in G3 (Fig. 3C). Deep myometrial invasion (> 50%) is an aggressive indicator of gynecological malignancies, often associated with advanced stages and poor outcomes. In cases of significant invasiveness (depth > 50%), *Porphyromonas*, *Fenollaria*, and *Moryella* were notably enriched (Fig. 3E ).

Among enrolled patients, 86.5% presented with chronic cervicitis, a common inflammatory disorder of the uterine area that may influence the microenvironment of endometrial carcinoma by altering microbial structures. LEfSe analyses showed significant enrichment of *Sphingomonas* and *Staphylococcus* in patients with chronic cervicitis (Fig. 3F). The Welch t-test confirmed this finding, revealing that several bacterial taxa, including *Staphylococcus*, *Cutibacterium*, and *Corynebacterium*, were particularly enriched in chronic cervicitis (*P* < 0.01; Fig. 3G).

### Invasion and pro-inflammatory capacity of cancer-derived strains

**Fig. 4:**
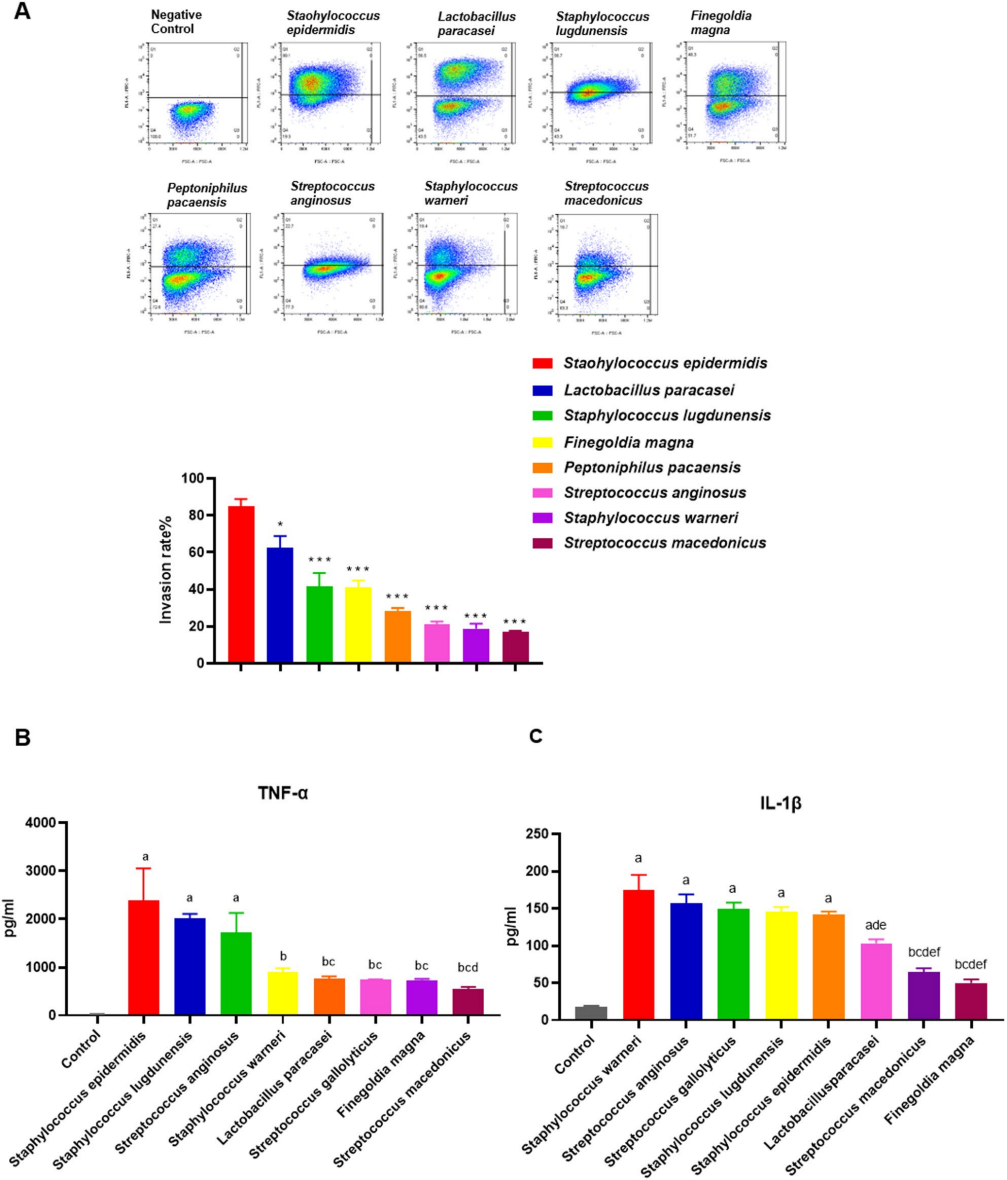
Assessment of invasion and pro-inflammatory capacity in cancer-derived strains. **A** Flow cytometry was used to determine bacterial invasive capacity. **B** TNF-α levels in cancer-derived strains with invasion efficiency ≥ 15%. **C **IL-1β levels in cancer-derived strains with invasion efficiency ≥ 15%. Control = a, *Staphylococcus epidermidis* = b, *Staphylococcus lugdunensis* = c, *Staphylococcus warneri* = d, *Streptococcus anginosus* = e, *Streptococcus gallolyticus* = f. If there are statistically significant differences between groups, different letters are used; otherwise, the same letters are used. *P* values were calculated using one-way ANOVA, with *P* value < 0.05

We further assessed the invasion efficiency of 31 species isolated from CATs using flow cytometry, revealing distinct hierarchical differences in invasion efficiency across these strains. *Staphylococcus epidermidis* (84.8%) displayed the highest invasion rate, followed by *Lactobacillus paracasei* and *Finegoldia magna* at approximately 50%, both of which also indicated relatively strong invasive potential (Fig. 4A). The majority of strains were classified as having low invasion efficiency, with *Veillonella parvula* (14.17%) and *Corynebacterium uberis* (13.57%) being the most prevalent. In contrast, the invasion efficiency of certain strains, such as *Enterococcus faecalis* (0.94%) and *Escherichia coli* (0.18%), was negligible (< 1%) (Figure S6). Subsequently, we analyzed the expression of inflammatory cytokines in strains with invasion efficiency ≥ 15%, corresponding to the 75th percentile by quartile analysis. Strains from the *Staphylococcus* and *Streptococcus* genera significantly increased the expression levels of TNF-α and IL-1β. *Staphylococcus epidermidis* showed the most significant increase, followed by *Staphylococcus lugdunensis*, *Streptococcus anginosus*, and *Staphylococcus warneri*.

## Discussion

This study comprehensively characterized the microbial community profiles of endometrial carcinoma using bacterial isolation and culture combined with 16S rDNA sequencing technology. The results revealed significant associations between microbial community characteristics and key clinicopathological features of EC, providing new insights into the potential role of the tumor microbiome in the occurrence and development of EC.

This study analyzed 32 patients with endometrial carcinoma. Consistent with clinical consensus, higher tumor grade correlated with greater malignant potential [21]. A significant age disparity was observed, supporting the established link between increased age and endometrial cancer risk, potentially mediated by age-related immune senescence and hormonal changes [22]. These host alterations may impact the local microbial community in a grade-dependent manner. Clinically, deep myometrial invasion (≥ 50%) was significantly associated with higher-grade tumors. Furthermore, LVSI—a key poor prognostic marker—was found exclusively in G3 tumors (40%), underscoring the link between high-grade, deep invasion, and metastatic potential [23]. This analysis confirms the expected correlations between histologic grade and clinicopathological features, providing a necessary clinical framework for interpreting subsequent microbial findings.

FISH analysis confirmed intratumoral bacteria in endometrial tissues, with stronger signals in cancerous areas, directly challenging the notion of a sterile uterus [24]. Through culturomics, we isolated and identified 79 bacterial species from vaginal swabs, CATs, and NATs. The phylum-level composition was similar across sites, with *Firmicutes* dominant (61.7%). While high-frequency isolates were typically shared among all three sites, uniquely or partially shared species were less frequent, highlighting distinct site-specific communities alongside a conserved cross-site core microbiota [25].To rigorously define cross-site core microbiota, we combined discrete random expectation and the quartile method, using the 75th percentile as the cutoff. Of all isolated bacterial species, 14 exhibited widespread distribution across the three sample types. With a cutoff value at 44.5%, 7 species were identified as cross-site core taxa, including *Staphylococcus epidermidis*, *Streptococcus anginosus*, *Finegoldia magna*, *Enterococcus faecalis*, *Escherichia coli*, *Staphylococcus lugdunensis*, and *Cutibacterium acnes*. At the genus level, 9 genera with random expectation ≥ 49.6% were defined as core members: *Staphylococcus*, *Streptococcus*, *Finegoldia*, *Enterococcus*, *Corynebacterium*, *Escherichia*, *Cutibacterium*, *Actinomyces*, and *Peptoniphilus*. Among these core taxa *Staphylococcus* showed the highest cross-site frequency at the genus level, with *Staphylococcus epidermidis* most common in vaginal swabs and CATs and *Finegoldia magna* in NATs. *Streptococcus anginosus* and *Enterococcus faecalis* also demonstrated high cross-site prevalence. *Cutibacterium* was notably prevalent, second only to *Staphylococcus* in CATs. The core genera identified here overlap with those reported by Leoni et al. using ddPCR, including *Corynebacterium* and *Cutibacterium* (100% detection) and *Staphylococcus* and *Streptococcus* (> 88% detection), reinforcing a link between these taxa and the tumor microenvironment [26]. Importantly, these genera have been documented to play roles in tumor progression. Tumor-associated *Staphylococcus* is associated with lung cancer metastasis through a pseudo-hypoxia signaling pathway [27], and *Staphylococcus epidermidis* has been shown to contribute to invasive progression in breast cancer [28]. Meanwhile, *Cutibacterium* species are enriched in prostate [29] and thyroid [30] cancers and have been linked to local immune suppression and a poorer prognosis. *Streptococcus* species are implicated in the development of gastric [31] and colorectal [32] cancer. Moreover, anaerobic species co-isolated from two sites, such as *Anaerococcus vaginalis* and *Peptoniphilus harei*, showed measurable isolation frequencies in vaginal specimens (21.1% and 31.6%, respectively). The genus-level analysis indicated that *Peptoniphilus* was classified into the nine cross-site core genera, while the cutoff value of *Anaerococcus* (41.2%) was slightly lower than core genera. Though *Anaerococcus* failed to meet the core threshold, it exhibited a colonization pattern similar to core taxa, consistent with microbial distribution characteristics of the female reproductive tract. The presence of these cross-site core microbial communities indicates that the cancer tissue microbiome originates from the vagina and adjacent normal tissues [17]. The unique enrichment of skin and oral commensal bacteria, such as *Staphylococcus capitis* and *Streptococcus salivarius*, in CATs provides a novel perspective on the pathogenesis of this disease from a microbiome standpoint. This phenomenon may not only indicate ectopic colonization pathways, such as the “oral-reproductive tract” axis, but also suggest that the tumor microenvironment selectively recruits and adapts to particular bacterial species. The comprehensive spectrum of microbial colonization, encompassing both shared and uniquely enriched bacteria, provides multifaceted insights into disease pathogenesis and lays a potential foundation for the development of microbiome-based diagnostic and therapeutic strategies [33].

16S rDNA sequencing revealed distinct differences in microbial community composition and diversity among the samples. *Lactobacilli* dominated the vaginal microbiota, whereas the endometrium showed a shift, with *Stenotrophomonas* as the dominant genus, highlighting a significant difference between vaginal and endometrial tissues [34]. This pattern aligns with the evolutionary progression from non-cancerous to cancerous states, in which the vaginal microbial community, initially dominated by *Lactobacilli*, is progressively supplanted by opportunistic pathogens [35]. *α*- and β-diversity analyses showed significant differences in species richness and microbial community structure in cancer tissue microbiota, with a substantial decrease in species richness compared with the other groups [36]. In comparing NATs and CATs, *Lactobacillus*, *Fenollaria*, and *Microbacterium* are notably more abundant in cancerous areas, whereas *Stenotrophomonas*, *Staphylococcus*, *Oceanimonas*, and *Cutibacterium* are more prevalent in adjacent sites. Although extensive research supports an inverse correlation between *Lactobacillus* and endometrial cancer, some studies suggest that specific strains may be associated with poor outcomes [37]. *L. iners* is strongly correlated with poor prognosis in cervical cancer, as it is associated with tumor radio- and chemoresistance through lactate-mediated metabolic reprogramming [38]. Studies have confirmed that, as opportunistic pathogens of the skin and mucosal organs, *Microbacterium* [39], *Stenotrophomonas* [40], and *Fenollaria* [41] elicit inflammatory responses, suggesting their potential role in shaping a pro-tumorigenic microenvironment.

This study provided evidence that distinct pathological statuses and clinical characteristics significantly are significantly associated with the uterine microbiome in patients with endometrial carcinoma. Notably, CATs exhibited greater species richness than paired NATs across all histological grades. Furthermore, significant differences in *β*-diversity were observed between CATs and vaginal samples across all histological grades. These results indicate that the tumor microenvironment exerts a specific modulatory effect on microbial community structure, consistent with prior research documenting alterations in the vaginal microbiome associated with histological grade of endometrial carcinoma [42]. Microbial enrichment exhibited grade-specific patterns; for instance, the genus *Slackia* was enriched in CATs. *Slackia* is commonly detected in human mucosal and gut microbiota, with some species involved in fatty acid and steroid metabolism [41], and it contributes to remodeling of the tumor microenvironment in gastric carcinogenesis [43]. The enrichment of *Porphyromonas* in poorly differentiated (G3) tumors and in tumors with deep myometrial invasion (> 50%) suggests its association with aggressive endometrial cancer phenotypes, as supported by existing research [12, 44].

In our study, 86.5% of enrolled patients had chronic cervicitis, with increases in genera such as *Staphylococcus*, *Cutibacterium*, and *Corynebacterium*, potentially altering the cancer-promoting environment by changing the microbial structure. This underscores the interaction between inflammation and microbial imbalance, supporting the finding that microbial dysbiosis contributes to the development of endometrial carcinoma by inducing chronic inflammation. The enrichment of pro-inflammatory genera in the endometrial microbiota may activate key signaling pathways, notably NF-κB, through pattern-recognition receptors such as TLR4 and NLRP3, thereby leading to significant upregulation of core pro-inflammatory cytokines, including IL-1β and TNF-α [14, 45]. These cytokines directly promote tumor cell proliferation, invasion, and angiogenesis while disrupting epithelial barrier integrity, creating a vicious cycle of “barrier disruption–chronic inflammation–tissue remodeling” that ultimately drives malignant transformation [46]. Furthermore, mediators such as IL-1β can polarize macrophages toward a pro-tumor M2 phenotype and suppress cytotoxic T cell function, thereby shaping an immunosuppressive tumor microenvironment [47, 48]. Our in vitro study revealed that strains with high invasive efficiency elicited a significant proinflammatory response. *Staphylococcus epidermidis* exhibited the highest invasion efficiency (84.8%), followed by *Staphylococcus lugdunensis* and *Streptococcus anginosus*, which also notably upregulated the expression of pro-inflammatory cytokines TNF-α and IL-1β. These findings suggest that highly invasive strains of *Staphylococcus* [49] and *Streptococcus* [50] may contribute to the development of EC through colonization, invasion, and induction of local chronic inflammation.

However, discrepancies between culturomics and 16S rDNA sequencing highlight the limitations of our study. Culturomics is limited to isolating only cultivable bacterial strains, potentially overlooking non-culturable microbial taxa in the tumor microenvironment, such as *Stenotrophomonas* and *Porphyromonas*, which were identified as dominant by 16S rDNA sequencing but were not isolated in our study. This discrepancy likely arises from enrichment culture conditions that do not meet the specific growth needs of these taxa, highlighting the limitations of culturomics in fully representing microbial communities. Furthermore, it remains challenging to correlate 16S rDNA sequencing data with culturomics data, and metagenomics will facilitate more precise integration of microbial abundance and functional phenotypes in future studies. This further demonstrates the need for multi-omics approaches to comprehensively characterize tumor-associated microbiota. While 16S rDNA sequencing provides a broad microbial profile, it lacks precise strain-level identification and viability assessment. The combined detection biases inherent in these two approaches led to a one-sided characterization of microbial community composition and functions in this study. Furthermore, the sample size of endometrial cancer tissues analyzed in this study was relatively limited, making it vulnerable to individual variability and insufficient to represent microbial community characteristics across various clinical subtypes, age groups, and genetic backgrounds. This limitation heightened the potential for bias in microbial detection and analysis. For instance, the observed LVSI rate of 40% in the G3 group (Table 1) might reflect an exaggerated difference due to the small subgroup size. Future research should involve multicenter collaboration to expand the cohort and perform stratified analyses on adequately sized subgroups, thereby improving the reliability and generalizability of the results. Additionally, our investigation was confined to assessing the invasive and proinflammatory capacities of the strains, without conducting comprehensive evaluations of their in vivo pro-tumor effects or the associated molecular regulatory mechanisms. Consequently, the findings of this study warrant further validation in future research.

In conclusion, this study examined the microbiome in cancerous tissues, non-cancerous adjacent tissues, and vaginal samples from patients with endometrial carcinoma, using both culturomics and 16S rDNA sequencing. Culturomics analysis identified *Staphylococcus* and *Streptococcus* as predominant cross-site core genera, characterized by high prevalence, robust invasive efficiency, and proinflammatory potential. These results suggest that dysbiosis of the reproductive tract microbiome, originating in the vaginal environment, may negatively impact the endometrium. Furthermore, 16S rDNA sequencing revealed that the distribution of certain microbial strains correlates with tumor grade and the extent of myometrial invasion. This study provides foundational data on microbial diversity in patients with endometrial carcinoma, identifying microorganisms that could serve as biomarkers and targets for diagnosis and treatment, and exploring potential pathogenic links using isolated strains.

## Supplementary Information

Supplementary Material 1.

## Data Availability

Raw sequencing data is available in the NCBI Sequence Read Archive (https://www.ncbi.nlm.nih.gov/sra/PRJNA1420213).

## Abbreviations

EC: Endometrial cancer
CATs: Cancerous tissues
NATs: Non-cancerous adjacent tissues
CTAB: Cetyltrimethylammonium bromide
CFSE: 5-(and 6)-carboxyfluorescein diacetate succinimidyl ester
