# Supplementary material for: Integrated culturomics and 16S rDNA sequencing reveal a functional microbiome signature in endometrial cancer
Source: BMC Microbiol. 2026 Jun 10;26:727. doi: 10.1186/s12866-026-05242-x (PMC13474856; doi:10.1186/s12866-026-05242-x)
Supplement: Supplementary file 1 [file 12866_2026_5242_MOESM1_ESM.docx]

Table S1. Clinical characteristics across different pathological groups

| **Category（Total=N/%）** | **Histological grade** | | | ***P-*value** |
| --- | --- | --- | --- | --- |
|  | **G1(N=12)** | **G2(N=15)** | **G3(N=5)** |  |
| CA125(15/46.9) | 5(41.7) | 8(53.3) | 2(40) | 0.624 |
| CA19-9(11/34.4) | 3(25) | 6(40) | 2(40) | 0.758 |
| ER (30/93.8) | 11(91.7) | 14(93.3) | 5(100) | 0.845 |
| PR (29/90.6) | 11(91.7) | 14(93.3) | 4(80) | 0.698 |
| MMR |  |  |  | 0.253 |
| dMMR(6/18.8) | 1(8.3) | 5(33.3) | 0(0) |  |
| pMMR(26/81.3) | 11(91.7) | 10(66.7) | 5(100) |  |
| Ki67 proliferation index |  |  |  | 0.867 |
| <10% (4/13.5) | 2(16.7) | 2(13.3) | 0(0) |  |
| 10%-50% (15/46.8) | 5(41.7) | 8(53.3) | 2(40) |  |
| >50% (13/40.6) | 5(41.7) | 5(33.3) | 3(60) |  |
| p53 mutation (5/15.6) | 3(25) | 1(6.7) | 1(20) | 0.616 |
| PAX-2 deficiency (31/83.8) | 10(83.3) | 12(80) | 4(80) | 0.758 |
| PTEN deficiency (26/82.3) | 8(66.7) | 12(80) | 4(80) | 0.854 |

Table S2. Species isolated exclusively from the CATs group

| **Species** | **Frequency of detection (%)** | **Random expectation (%)** |
| --- | --- | --- |
| *Staphylococcus capitis* | 10.5 | 10.5 |
| *Streptococcus salivarius* | 5.3 | 5.3 |
| *Streptococcus macedonicus* | 5.3 | 5.3 |
| *Bacillus infantis* | 5.3 | 5.3 |
| *Veillonella parvula* | 5.3 | 5.3 |
| *Cutibacterium granulosum* | 5.3 | 5.3 |
| *Corynebacterium sp. MC3* | 5.3 | 5.3 |
| *Brevibacterium ammoniilyticum* | 5.3 | 5.3 |

Table S3. Bacterial species isolated from any two sample types

| **Isolatied sites** | **Species** | **Frequency of detection (%)** | | | **Random expectation (%)** |
| --- | --- | --- | --- | --- | --- |
|  |  | **Vaginal swab(N=19)** | **NATs(N=19)** | **CATs(N=19)** |  |
| NATs and CATs | *Staphylococcus pasteuri* | 0 | 5.3 | 5.3 | 10.3 |
|  | *Staphylococcus caprae* | 0 | 5.3 | 5.3 | 10.3 |
|  | *Dialister invisus* | 0 | 5.3 | 5.3 | 10.3 |
|  | *Lactobacillus paracasei* | 0 | 5.3 | 5.3 | 10.3 |
|  | *Bacteroides vulgatus* | 0 | 5.3 | 10.5 | 15.2 |
| Vaginal and CATs | *Staphylococcus warneri* | 5.3 | 0 | 5.3 | 10.3 |
|  | *Anaerococcus vaginalis* | 21.1 | 0 | 5.3 | 25.3 |
|  | *Actinomyces neuii* | 5.3 | 0 | 5.3 | 10.3 |
|  | *Actinomyces turicensis* | 5.3 | 0 | 5.3 | 10.3 |
| Vaginal and NATs | *Staphylococcus aureus* | 5.3 | 5.3 | 0 | 10.3 |
|  | *Peptoniphilus lacrimalis* | 10.5 | 5.3 | 0 | 15.2 |
|  | *Peptoniphilus harei* | 31.6 | 10.5 | 0 | 38.8 |
|  | *Bacteroides fragili* | 10.5 | 5.3 | 0 | 15.2 |

Figure S1 Workflow chart of sample collection processing.

Figure S2. Proportion of isolated species at the phylum level.

Figure S3. Endometrial and vaginal microbiota *α*-diversity. (A) Shannon diversity index for endometrial and vaginal samples. (B) Simpson diversity index for endometrial and vaginal samples.

Figure S4. *β*-diversity in vaginal (A) and NATs (B) across histological grades. Bray-Curtis *β*-diversity distance matrix presented as a box plot and PCoA.

Figure S5. *α*-diversity in CATs among histological grades. (A) Chao1 diversity index. (B) Shannon diversity index. (C) Simpson diversity index.

Figure S6. Assessment of invasion capacity in cancer-derived strains. Flow cytometry was used to determine bacterial invasive rates ranging from 1%–15% (A) to less than 1% (B).
